# Early Economic Evaluation Demonstrates That Noncomputerized Tomography Robotic-Assisted Surgery Is Cost-Effective in Patients Undergoing Unicompartmental Knee Arthroplasty at High-Volume Orthopaedic Centres

**DOI:** 10.1155/2020/3460675

**Published:** 2020-04-14

**Authors:** Leo M. Nherera, Sanjay Verma, Paul Trueman, Simon Jennings

**Affiliations:** ^1^Health Economics and Market Access, Smith & Nephew, 5600 Clearfork Main St, Fort Worth, TX 76107, USA; ^2^Health Economics and Market Access, Smith & Nephew, 5 Croxley Green Business Park, Hatters Ln, Watford WD18 8YE, Watford, UK; ^3^Health Economics and Market Access, Smith & Nephew, 101 Hessle Rd, Hull HU3 2BN, UK; ^4^London North West University Healthcare NHS Trust, Acton Lane Park Royal, London NW10 7NS, UK

## Abstract

**Background:**

For over fifty years, unicompartmental knee arthroplasty (UKA) has been used to treat single-compartment osteoarthritis of the knee and is considered a safe alternative to total knee arthroplasty (TKA). The development and use of robotic-assisted surgery (r-UKA) have made the execution of the procedure more precise, and various studies have reported improved radiographic outcomes and implant survival rates; however, its cost-effectiveness is unknown. This study aimed at assessing the cost-effectiveness of noncomputerized tomography (non-CT) r-UKA compared to the traditional unicompartmental knee arthroplasty (t-UKA) method in patients with unicompartmental knee osteoarthritis from the UK payer's perspective.

**Methods:**

We developed a 5-year four-state Markov model to evaluate the expected costs and outcomes of the two strategies in patients aged 65 years. Failure rates for t-UKA were taken from the British National Joint Registry while data for non-CT r-UKA were obtained from a 2-year observational study. Cost was obtained from the NHS reference cost valued at 2018/19 GBP£, and a discount rate of 3.5% was applied to both costs and benefits.

**Results:**

For a high-volume orthopaedic centre that performs 100 UKA operations per year, non-CT r-UKA was more costly than t-UKA but offered better clinical outcomes, and the estimated cost per QALY was £2,831. The results were more favourable in younger patients aged less than 55 and sensitive to case volumes and follow-up period.

**Conclusion:**

Non-CT r-UKA is cost-effective compared with t-UKA over a 5-year period. Results are dependent on case volumes and follow-up period and favour younger age groups.

## 1. Introduction

For over fifty years, unicompartmental knee arthroplasty (UKA) has been used to treat single-compartment osteoarthritis of the knee and is considered a safe alternative to total knee arthroplasty (TKA) [1, 2]. There are a number of documented surgical benefits associated with UKA when compared with TKA which include less perioperative morbidity, reduced blood loss, and shorter postoperative recovery and rehabilitation [3–5]. A recent systematic review and meta-analysis using data from randomised controlled trials, observational studies, and registry studies reported shorter mean length of stay, better functional patient-reported outcome measure scores, and fewer revisions compared to TKA [6].

UKA procedure volumes are low compared to TKA with suggestions that adoption is limited by perceived technical difficulty in performing the procedure and concerns over the variation in outcomes in the published literature [7, 8]. Failures of UKA have been attributed to medial-lateral mismatch, inadequate stability of the components, and improper alignment [7, 9]. The development and use of robotic-assisted surgery (r-UKA) have made the execution of a technically difficult procedure more precise, and various studies have reported improved radiographic outcomes and in particular improved implant alignment [10–14]. It is estimated that approximately 14% of US UKA surgeries were performed with robotic assistance as of 2012 and this rate is expected to increase [11]. Studies have also observed an association between high-volume centres and better outcomes, in particular revision rates [15–17].

The capital costs of robotic assistance mean that a crude comparison of procedural costs of traditional instrumentation versus robotic-assisted UKA would favour the traditional procedure [12]. However, it is important that the incremental costs associated with robotic-assisted surgery consider potential impacts on clinical outcomes. Cost-effectiveness analyses can help to reduce decision uncertainty by allowing a comparative analysis of both the costs and health outcomes (revisions avoided) of traditional unicompartmental knee arthroplasty (t-UKA) versus non-CT r-UKA. Our study, therefore, aimed at evaluating the cost-effectiveness of non-CT r-UKA when compared to that of t-UKA in the UK. Whilst the evidence on non-CT r-UKA remains limited at the current time, early economic modelling allows for the cost-effectiveness to be estimated based on the available evidence and through extrapolation of outcomes. Over time, early models can be validated as further data are made available.

## 2. Materials and Methods

### 2.1. Overview and Modelling Framework

**Figure 1:**
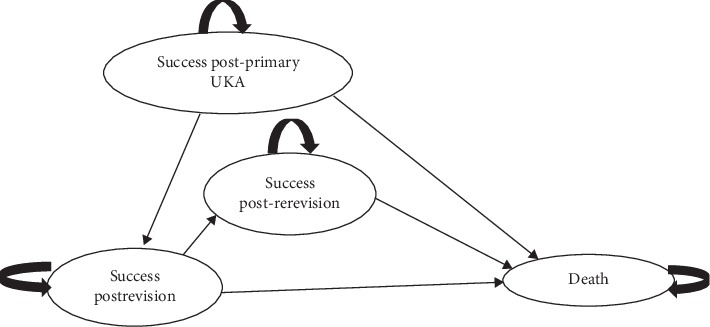
Schematic representation of the model structure.

A Markov model with four health states was developed in Microsoft® Excel from the perspective of UK National Health Service payer. The four discrete health states following primary UKA surgery were as follows: (1) successful primary UKA, (2) revision UKA, (3) rerevision UKA, and (4) death.  Figure 1 shows the diagrammatic representation of the model structure.

The model considers a cohort of 100 patients with a mean age of 65 years who are eligible for primary UKA. This age group was considered to be broadly representative of a typical patient for UKA surgery according to registry data [7, 18].

The model estimates the costs and outcomes of treating 100 patients managed with either non-CT r-UKA (Navio Smith & Nephew, Memphis, TN) compared with t-UKA. Given limitations in the data, the model recognises the difference in surgical approach but makes no attempt to distinguish the effectiveness of particular implant designs. Following primary UKA surgery with or without robotic assistance, patients can enter a successful primary UKA state, but remain at risk of entering a revision surgery state due to UKA failure, i.e., all-cause revision. For patients that transition into the revision surgery state, these patients can enter into a successful postrevision state or are at further risk of transition to a rerevision surgery state. No further surgical options were considered beyond a second revision (rerevision), as we assumed patients will be converted to total knee arthroplasty. Lastly, patients may transition to the death state from any other health state and experience no further transitions. Individuals in the model can transition between the health states each year. The model has a 5-year time horizon. The model estimates the number of individuals in each health state every year, considering the risk of health states worsening (e.g., failure).

### 2.2. Clinical Data Inputs for Traditional UKA and Robotic-Assisted Surgery

Clinical data on traditional UKA were derived from the 2018 National Joint Registry [18] from England and Wales. The NJR was set up in 2002 to collect information on joint replacement operations and performance of implants and surgeons. The failure rate of all UKA surgeries as defined by revisions at 5 years was reported to be 6.1%. Revision was defined as a reoperation resulting in the modification of the primary UKA or conversion to TKA. Rerevision was defined as a reoperation of a revised UKA [19]. Due to the low incidence of revision surgery, a one-year cycle length was utilized and is in consistent with published studies [19, 20]. The following formula was used to convert the reported 5-year rates to annual probabilities for use in the model [21].

(1) $$\begin{array}{c} p\;=1-\;e^{-rt}, \end{array}$$

where *p* is the probability, *r* is the rate, and *t* is the unit of time.

Clinical data on the performance of non-CT r-UKA were obtained from a retrospective multicenter, cohort study on patients who had undergone robotic-assisted surgery with 2.3 years of follow-up [7]. Given the limited follow-up data, it was necessary to extrapolate outcomes to 5 years to allow for comparison with NJR data. We adopted a conservative assumption on the effect of robotic surgery on revision rates. We assumed that any incremental benefit of non-CT r-UKA occurs in the first two years in accordance with the available clinical trial data. After this time, the risk of revision surgery was assumed to be the same as traditional UKA from year 3 to 5.

Overall survivorship of the UKA knee implants was 99.2% (95% confidence interval: 94.6% to 99.9%) [7]. These data were used to derive the hazard rate (HR) of revision surgery with non-CT r-UKA compared to t-UKA. Registry data on t-UKA identified 95,836 UKAs, of which 2863 needed a revision at 2 years [18]. This is compared to 1 revision in 128 knees seen in non-CT r-UKA study. The HR was therefore calculated to be 0.20 (95% CI: 0.17 to 0.24; *p* < 0.05). The HR was applied only for the first two years, after which we assumed the revision rates of non-CT r-UKA to be similar to that of t-UKA.

### 2.3. Mortality Rates

The all-cause age-specific mortality was obtained from UK Life Tables 2015/17 [22] (Table 1). For the base model, 5-year mortality rates following surgery were used. We assumed that both primary non-CT r-UKA and t-UKA had no impact on mortality; hence, similar mortality rates were experienced in both groups of patients. We made a further assumption that the probability of death after revision surgery due to UKA was the same in both the non-CT r-UKA- and t-UKA-treated patients.

### 2.4. Health-Related Quality of Life (HRQOL) and Utility Data Used in the Model

Outcomes of the analysis are presented as cost per revision avoided and cost per quality-adjusted life year (QALYs). QALYs are a measurement of quality of life defined on a scale of 0–1. A year of perfect health is equivalent to 1 QALY while a year of less than perfect health is worth less than 1 QALY. This outcome is widely used by health technology assessment bodies (such as NICE) as it generates a common currency which can be used to assess the cost-effectiveness of any health technology. A benchmark of £20,000–£30,000 per QALY is widely considered to represent a cost-effective use of healthcare resources in the NHS. The QALY for each health state is calculated by multiplying the health state utility (revision surgery, healed) by the model life span using the roll-back method. Utility data used were obtained from the published literature [19].

### 2.5. Case Volume and Healthcare Resource Costs

As robotic surgery requires capital expenditure, the cost per case will be dependent on procedural volumes. A previous study defined a high-volume arthroplasty centre as one which conducts >12 UKA cases per year or >200 TKA cases annually [19]. In the base case model, we considered a high-volume orthopaedic centre which conducts 100 UKA procedures per annum, similar to a previously published study of robotic surgery [19]. A break-even analysis was performed to evaluate the minimum surgical volume required to achieve cost-neutral non-CT r-UKA utilization and this issue was further assessed in sensitivity analyses.

The model adopted a payer perspective—considering only those costs and benefits that are incurred by the health service (the UK National Health Services) or the insurer (Table 1). Both future costs and benefits were discounted by 3.5% annually in accordance with the recommendations of the NICE reference case [24].

Costs included in the model were measured in GBP Pound sterling and were obtained from NHS reference costs [23, 25]. The reference costs include the procedure, implant, theatre, physician, and anaesthetic among other costs. The capital cost of CT-image free robotic system was estimated to be £358,000, which is the list price in the UK. This was adjusted in sensitivity analyses. Using the information on the assumed device life span (5 years) and discount rate (3.5%), we calculated the discounted annual equivalent costs (present value) of £79,290. In addition to the purchase price, the robot requires an annual service contract fee after the first year of ownership reported to be £21,500 per annum by the manufacturer. The annual equivalent costs, the annual service contract costs, and consumables were then summed and divided by the expected case volume of 100 cases per year to determine the cost per case of the robotic equipment. This cost was estimated to be £1,225 per case. Procedure costs were estimated as a weighted average from National Health Service (NHS) reference costs based on the relevant healthcare resource group (HRG) codes presented in Table 1.

### 2.6. Cost-Effectiveness Analysis and Sensitivity Analysis

The incremental cost-effectiveness ratio (ICER) is the added cost per additional unit of health benefit in this model measured as revisions avoided and QALYs. This is calculated as the difference between the expected costs of non-CT r-UKA and t-UKA divided by the difference between the expected numbers of QALYs between the two strategies over 5 years:

(2) $$\begin{array}{c} \text{ICER}=\frac{\Delta \text{cost}}{\Delta \text{QALYs}}. \end{array}$$

A one-way and probabilistic sensitivity analysis (SA) was implemented to evaluate the impact of uncertainty around the underlying data on base case model conclusions. One-way SA was implemented by varying some of the model parameters one at a time using lower and upper values reported in the literature and assessing the impact this had on model conclusions. Values were varied ±20% if ranges were not reported in the literature in accordance with other published economic studies. Furthermore, structural sensitivity analyses were conducted to assess the uncertainty of structural assumptions such as age, volume of cases seen, and time horizon which may affect outcomes. As age is a key variable in determining rates of revision surgery, we considered patient cohorts aged <55 years, 65–74 years, and >75 years to estimate the impact on outcomes. Probabilistic sensitivity analysis (PSA) was implemented by assigning statistical distributions to input parameters and vary the inputs simultaneously to assess the impact on model results. Clinical data inputs were assigned the lognormal distribution while the cost and utility data were assigned the gamma and beta distribution, respectively. The results of the PSA are presented on a cost-effectiveness acceptability curve. The curve shows the probability that each intervention is cost-effective at different willingness-to-pay (WTP) values. The WTP value is what the payer is prepared to pay for an additional unit of clinical benefit in this model measured in QALYs.

## 3. Results

### 3.1. Base Case Results

We report the results of a high-volume centre that performs 100 UKA procedures per year. Non-CT r-UKA was more costly than t-UKA but offered better clinical outcomes (there were fewer revisions and more QALYs) as shown in Table 2, and the estimated cost per QALY was £2,831.

### 3.2. Structural and One-Way Sensitivity Analysis

The base results are reported for a 65-year-old cohort of both males and females. In a separate analysis, we considered different age groups and non-CT r-UKA remained cost-effective across all age groups. However, the results were more favourable for the younger age group aged <55 years compared to those aged over 75 years. Gender, however, demonstrated only modest differences between the two cohorts (ICER male: £3,374; ICER female: £2,332/QALY). Extending the follow-up period beyond 5 years improved the cost-effectiveness of non-CT r-UKA, with it becoming cost saving with a follow-up beyond 7 years. One-way sensitivity analysis results suggest non-CT r-UKA is not sensitive to changes in key assumptions of revision probability, non-CT r-UKA effectiveness, and discount rate. Changes in these input parameters showed small changes in the estimated ICER, suggesting the reliability of the initial model assumptions. Sensitivity analysis results are summarised in Table 3.

**Figure 2:**
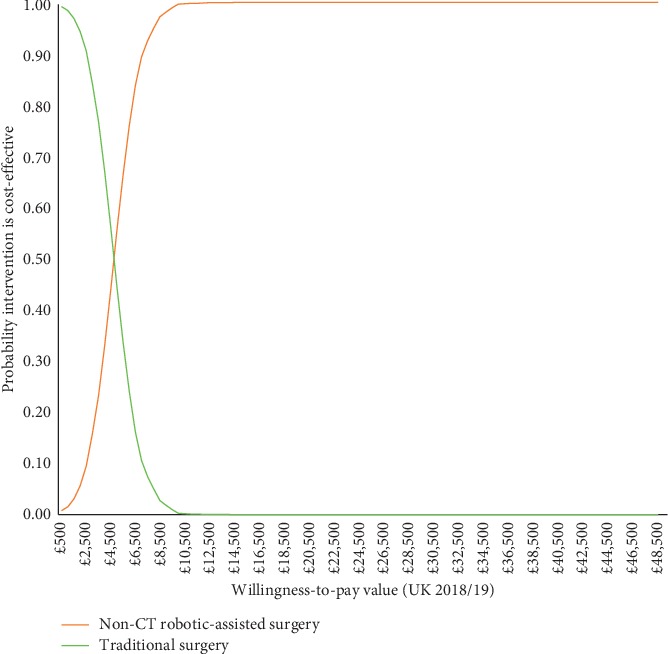
Cost-effectiveness acceptability curves.

The probability that non-CT r-UKA is cost-effective at a willingness-to-pay figure of £20,000/QALY is 100% as shown by the cost-effectiveness acceptability curve in Figure 2.

### 3.3. Number of Cases and Break-Even Analysis

**Figure 3:**
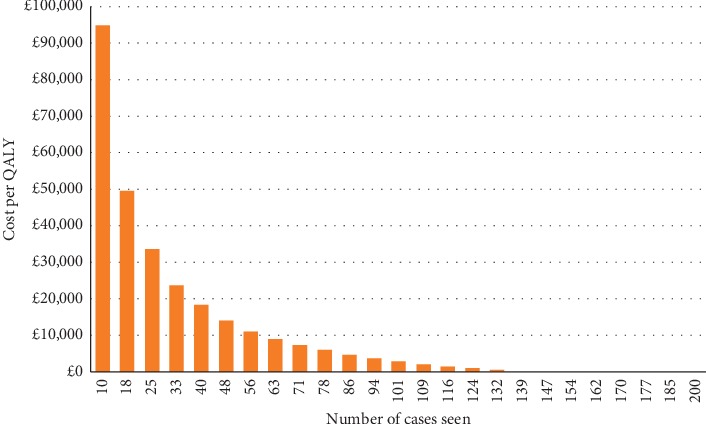
Number of procedures and the resultant cost per QALY.

As the cost per case is dependent on volume, we also varied the number of UKA procedures conducted to determine the impact on the cost-effectiveness ratio. Unsurprisingly, increasing the volume of procedures reduces the cost per case of robotic-assisted surgery and in doing so, improved the cost-effectiveness. Therefore, for high-volume facilities, the cost-effectiveness of non-CT r-UKA is favourable while for low-volume facilities the cost-effectiveness is less favourable. For example, for a centre that sees 20 patients per year, the cost per QALY is estimated to be £43,581, while for those that see more than 138 patients, non-CT r-UKA becomes cost saving compared to traditional UKA (see Figure 3).

## 4. Discussion

We assessed the cost-effectiveness of non-CT r-UKA compared to t-UKA from a UK health service perspective. Our base case analysis showed that non-CT r-UKA offered better clinical outcomes (fewer revisions) and hence more QALYs at an increased cost. The estimated cost per QALY is £2,831 for a high-volume facility that sees 100 patients per year, which is well within what is considered cost-effective in the NHS. This result is unlikely to be a chance finding as confirmed by the probabilistic sensitivity analysis.

Our study is the first of its kind comparing the cost-effectiveness of non-CT r-UKA with t-UKA based on clinical data from the UK. A previous study of robotic-assisted surgery for UKA based on data from the United States generated a cost-effectiveness ratio of $47,180 per QALY, significantly more than the current analysis. Whilst both studies adopted a cohort of 100 patients and based their analyses on 2-year follow-up data on robotic-assisted UKA, there were some notable differences in the methodologies and data sources. The differences result from a number of variables including higher procedural costs in the United States, differences in the time horizon adopted in the analyses, and differences in the robotic surgical systems considered.

Currently, the uptake of UKA is estimated to be less than 10% of total arthroplasties, yet UKA has been found to offer better clinical outcomes at lower costs when compared to TKA [26–28]. Cost savings have been attributed to reduced length of stay and reduced use of healthcare services, such as fewer outpatient visits in the first 2 years after the index surgery [27]. However, there is also a relationship between procedure volume and outcomes. A systematic review that compared TKA with UKA noted that centres that perform 20% of their arthroplasty practice as UKA achieve lower rates of revision for unexplained pain or failures [6]. It is speculated that one of the reasons low-volume centres observe higher failure rates of UKA is poor patient selection where surgeons are more likely to offer UKA to patients with partial-thickness cartilage loss which is associated with poor outcomes compared to patients with bone-on-bone arthritis [26, 27].

Our study corroborates this finding, indicating that non-CT r-UKA is cost-effective in centres conducting more than 30 non-CT r-UKA procedures per year and this improves dramatically in centres performing over 100 cases per year. Increasing the volume of non-CT r-UKA surgery and the frequency of procedures for individual surgeons ensures that surgeons reduce the learning curve, maintain their knowledge of the procedure, and ultimately, deliver improved outcomes [6, 29].

Robotic-assisted surgery has the potential to reduce variation in practice and promote more consistent outcomes in UKA, thereby enabling a shift in case mix and more widespread use of UKA for appropriate patients. This, in turn, may allow for more rapid recovery than TKA procedures and a shorter hospital stay. However, it is vital that surgeons have access to appropriate training on non-CT r-UKA and increase their procedure volume to maintain familiarity with the approach.

The current analysis should be considered as an “early” economic evaluation of robotic-assisted UKA, acknowledging that there are currently limited data on the effectiveness of this approach, particularly in terms of patient follow-up. In recognition of this, we made a number of conservative assumptions such as limiting the impact of non-CT r-UKA on revisions to 2 years following surgery and assumed that non-CT r-UKA had no impact on rerevisions and quality of life. These assumptions have the potential of underestimating the true benefits of non-CT r-UKA compared to t-UKA. However, we cannot quantify this benefit due to the lack of information in the published studies that were included in the analysis.

We also derived baseline rates of revision for UKA from registry data. Whilst these data are widely used for evaluations of this sort, they provide a mean rate which includes data from high- and low-volume surgeons. Given the complexity of UKA, it seems likely that high-volume surgeons may have better outcomes which would alter the findings of this analysis. We have attempted to address his through sensitivity analyses around the hazard rate although further research is required to generate comparable cohorts of patients and surgeons.

We conducted a number of one-way sensitivity analyses which demonstrated that the model was sensitive to case volume. Centres with higher case volume are bound to benefit more from non-CT r-UKA since the cost per case falls as the capital is deployed across greater numbers of patients. The model was based on an assumed cohort of 100 patients, as is typical in studies of this type. However, we recognise that the throughput of UKA is currently below this in a significant number of facilities. Sensitivity analyses illustrate the importance of volume and emphasise that robotic-assisted surgery will be more cost-effective in facilities that are designed to maximise their use of the capital equipment.

The model is also sensitive to assumptions around follow-up for instance if the model is analysed over 2 years, the estimated cost per QALY increases to £33,704 while if the model is analysed over 7 years, the model becomes cost saving. Ultimately, this issue can only be addressed through further long-term follow-up of robotic-assisted UKA patients to determine whether the short-term outcomes are sustained or even improve relative to traditional UKA.

Economic evaluation should be an iterative process and refined as uncertainty around the model parameters decreases. Our study is supported by robust clinical evidence based on a single multicentre retrospective cohort study with 2.3 years' worth of survivorship data in 128 patients. We appreciate these data are indicative of the potential benefits of non-CT r-UKA and that the analysis would need to be refined as more data become available. “Early” economic models are intended to inform decision making and investments early in the life cycle of promising technologies. Healthcare providers have the option of being risk-averse and delaying investment decisions indefinitely until uncertainty is addressed, or more assertive in their adoption of new technologies. In the case of orthopaedic interventions, where 5- and even 10-year outcomes are considered to be the gold standard of evidence, this is challenging. Delaying a decision on the adoption of a new technology for 10 years may reduce the risk of a bad investment but also deprives patients' access to promising technologies. Furthermore, by the time evidence is available, further technological advances are likely to have occurred. Early economic models, which incorporate sensitivity analyses to identify critical uncertainties, can help decision makers to make more informed choices about if and when they adopt promising innovative technologies.

## 5. Conclusion

According to our economic analysis, the use of non-CT robotics-assisted UKA may be more cost-effective when compared to traditional UKA for patients with knee osteoarthritis when performed at high-volume orthopaedic centres with experienced clinicians. These findings remained robust when different assumptions were tested. Future studies with longer time horizons are warranted to elucidate what benefits may persist beyond the reported two years and also to validate these preliminary findings. Clinicians and policy makers should consider adopting robotics as they strive to provide high-quality, value-based healthcare. This will allow the generation of more clinical data necessary to perform a definitive economic analysis in the near future.

## Figures and Tables

**Table 1:** Model input parameters.

*Revision data at 5 years converted to annual probabilities*				
Age	Mean	95% LCI	95% UCI	Reference
65	1.19%	1.15%	1.22%	[18]

*Rerevision all ages*				
All	2.05%	1.89%	2.21%	[18]

*Robotic-assisted UKA effectiveness*				
	Hazard rate	95% LCI	95% UCI	
Revision	0.2000	0.1700	0.2400	[7]

*Mortality following surgical procedure*				
Age	Mean	95% LCI	95% UCI	
65	4.44%	4.42%	4.47%	[18]

*All-cause mortality*				
Age 65	4.9%			[22]

*Quality of life weights*				
Postsurgery	0.750	0.600	0.938	[19]
Revision	0.565	0.452	0.706	[19]
Rerevision	0.4630	0.370	0.579	[19]
Mortality	0.000	0.000	0.000	[19]

*Costs GBP £2018/19*				
Revision/rerevision	£10,390	£7,793	£12,988	[23]
Arthroplasty	£6,267	£4,700	£7,833	[23]
Rehabilitation	£289	£217	£361	[23]
Consumables (burr, drape, discs)	£260	£195	£325	Manufacturer
Robotics costs	£358,000	£286,400^*∗*^	£429,600^*∗*^	Manufacturer
Annual service contract (year 2–5)	£21,500			Manufacturer

NJR = National Joint Registry of England and Wales; LCI = lower value of the 95% confidence interval; UCI = upper value of the 95% confidence interval; ^*∗*^assumed ±20%.

**Table 2:** Cost-effectiveness results of non-CT r-UKA compared to t-UKA for 100 treated patients.

Intervention	Costs	Number of revisions avoided	Cumulative QALYs	Incremental costs	Complications avoided	Cost/complication avoided	Difference in QALYs	Cost/QALY
Traditional UKA	£853,034	86	431					
Robotic-assisted UKA	£879,852	96	440	£26,8178	11	£2,521	9.47	£2,831

**Table 3:** Structural and one-way sensitivity analysis.

Parameter	Cost per QALY
Base case	**£2,831**
All age groups	£3,353
Age <55	Non-CT r-UKA dominating
Age 65–74	£6,578
Age >75	£10,283
Males	£3,373
Females	£2,332
2-year follow-up	£33,704
7-year follow-up	Non-CT r-UKA dominating
Revision probability lower value	£3,686
Revision probability upper value	£2,039
Non-CT r-UKA effectiveness lower value	£2,358
Non-CT r-UKA effectiveness upper value	£3,520
Discount rate lower value	£690
Discount rate upper value	£2,831
Cost of robotics/case lower value	£1,157
Cost of robotics/case upper value	£5,676

Non-CT r-UKA: noncomputerized tomography robotic-assisted unicompartmental knee arthroplasty.

## Data Availability

The clinical and cost data used to support the findings of this study are included within the article.
